# Evaluation of intensive community care services for young people with psychiatric emergencies: study protocol for a multi-centre parallel-group, single-blinded randomized controlled trial with an internal pilot phase

**DOI:** 10.1186/s13063-024-07974-5

**Published:** 2024-02-22

**Authors:** Thilipan Thaventhiran, Ben Hoi-Ching Wong, Izabela Pilecka, Saba Masood, Opeyemi Atanda, Joe Clacey, Jovanka Tolmac, Leon Wehncke, Liana Romaniuk, Margaret Heslin, Emma Tassie, Petrina Chu, Rhys Bevan-Jones, Ruth Woolhouse, Tauseef Mahdi, Veronika Beatrice Dobler, Mandy Wait, Paula Reavey, Sabine Landau, Sarah Byford, Toby Zundel, Dennis Ougrin

**Affiliations:** 1 Queen Mary University of London, London, UK; 2 East London NHS Foundation Trust, London, UK; 3 King’s College London, London, UK; 4 London South Bank University, London, UK; 5 Oxford Health NHS Foundation Trust, Oxford, UK; 6 Central and North-West London NHS Foundation Trust, London, UK; 7 North-East London NHS Foundation Trust, London, UK; 8 NHS Lothian, Edinburgh, UK; 9 Cwm Taf Morgannwg University Health Board, Wales, UK; 10 Cardiff University, Wales, UK; 11 Berkshire Healthcare NHS Foundation Trust, Bracknell, UK; 12 Cambridgeshire and Peterborough Foundation Trust, Cambridge, UK; 13 South London and Maudsley NHS Foundation Trust, Beckenham, UK

**Keywords:** Young people, Children, Community care, Intensive community care services, Inpatient care, Mental health

## Abstract

**Background:**

Over 3000 young people under the age of 18 are admitted to Tier 4 Child and Adolescent Mental Health Services (CAMHS) inpatient units across the UK each year. The average length of hospital stay for young people across all psychiatric units in the UK is 120 days. Research is needed to identify the most effective and efficient ways to care for young people (YP) with psychiatric emergencies. This study aims to evaluate the clinical effectiveness and cost-effectiveness of intensive community care service (ICCS) compared to treatment as usual (TAU) for young people with psychiatric emergencies.

**Methods:**

This is a multicentre two-arm randomized controlled trial (RCT) with an internal pilot phase. Young people aged 12 to < 18 considered for admission at participating NHS organizations across the UK will be randomized 1:1 to either TAU or ICCS. The primary outcome is the time to return to or start education, employment, or training (EET) at 6 months post-randomization. Secondary outcomes will include evaluations of mental health and overall well-being and patient satisfaction. Service use and costs and cost-effectiveness will also be explored. Intention-to-treat analysis will be adopted. The trial is expected to be completed within 42 months, with an internal pilot phase in the first 12 months to assess the recruitment feasibility. A process evaluation using visual semi-structured interviews will be conducted with 42 young people and 42 healthcare workers.

**Discussion:**

This trial is the first well-powered randomized controlled trial evaluating the clinical and cost-effectiveness of ICCS compared to TAU for young people with psychiatric emergencies in Great Britain.

**Trial registration:**

ISRCTN ISRCTN42999542, Registration on April 29, 2020

## Administrative information

Note: the numbers in curly brackets in this protocol refer to SPIRIT checklist item numbers. The order of the items has been modified to group similar items (see http://www.equator-network.org/reporting-guidelines/spirit-2013-statement-defining-standard-protocol-items-for-clinical-trials/).

**Table:** 

Title {1}	Comparison of effectiveness and cost-effectiveness of intensive community care services versus treatment as usual including inpatient care for young people with psychiatric emergencies (IVY): an internal pilot followed by a randomized controlled trial comprising all Intensive community service care teams in Great Britain. Protocol Short title: Evaluation of Intensive Community Care Services for young people with psychiatric emergencies (IVY).
Trial registration {2a and 2b}	ISRCTN: ISRCTN42999542
Protocol version {3}	Version 2.7; August 10, 2023
Funding {4}	National Institute for Health and Care Research (NIHR) Health Technology Assessment (HTA) Programme. (Ref: NIHR127408).
Author details {5a}	^1^ Queen Mary University of London, London, UK ^2^ East London NHS Foundation Trust, London, UK ^3^ King’s College London, London, UK ^4^ London South Bank University, London, UK ^5^ Oxford Health NHS Foundation Trust, Oxford, UK ^6^ Central and North-West London NHS Foundation Trust, London, UK ^7^ North-East London NHS Foundation Trust, London, UK ^8^ NHS Lothian, Edinburgh, UK ^9^ Cwm Taf Morgannwg University Health Board, Wales, UK ^10^ Cardiff University, Wales, UK ^11^ Berkshire Healthcare NHS Foundation Trust, Bracknell, UK ^12^ Cambridgeshire and Peterborough Foundation Trust, Cambridge, UK ^13^ South London and Maudsley NHS Foundation Trust, Beckenham, UK
Name and contact information for the trial sponsor {5b}	King’s College London Name of Sponsor Representative: Professor Bashir Al-Hashimi Address: Room 8.11, 8th Floor Melbourne House, 44-46 Aldwych London WC2B 4LL. Telephone: 02078487306 Email: vpri@kcl.ac.uk South London and Maudsley NHS Foundation Trust Name of Sponsor Representative: Christina Armoogum Address: R&D Department, Room W1.08, Institute of Psychiatry, Psychology & Neuroscience (IoPPN), De Crespigny Park, London SE5 8AF Telephone: 020 784 80339 Email: slam-ioppn.research@kcl.ac.uk
Role of sponsor {5c}	Role of sponsor includes local Research and Development approval, institutional indemnity insurance for the trial, but does not include funding or trial conduct

## Introduction

### Background and rationale {6a}

A 2022 National Health Service (NHS) survey of children and young people’s (CYP) mental health in England found that around 1 in 6 children aged 5–16 years were identified as having a probable mental health problem [1]. The latest review of CAMHS (which include specialist and inpatient services) revealed that there were over 1.2 million CYP aged between 0 and 18 years referred for mental health support in 2021–2022, an increase of 41% since 2010–2021 [2]. Inpatient admissions for CYP can play an important role in providing intensive levels of care to stabilize severe mental health disturbances [3]. However, they can also be lengthy with interpersonal disconnection [4]. This is especially true for CYP who experience repeated admissions [5]. Over 1000 CYP have been placed ‘out of area’ for mental health care in England each year for the past 3 years, most detained under the Mental Health Act [6]. From 2021 to 2022, there was a 32% increase in the number of under-18s admitted to adult psychiatric wards in England, with 260 admissions compared to 197 in the previous year. The main reason for these admissions was a lack of alternative mental health inpatient or outreach services for CYP [7]. Inpatient admissions are more costly for healthcare systems than outpatient care. Demand can also outstrip capacity, resulting in admissions of CYP in adult psychiatric wards or non-mental health inpatient settings [8].

The COVID-19 pandemic has seen a worldwide increase in psychiatric emergencies [9], exacerbating the national bed crisis in the UK. The average length of hospital stay in the UK is 74 days for general admissions, 103 days for eating disorders units, and 307 days for secure units, which is longer than in many other developed countries [10]. A key contributor to the long length of hospital stay for CYP with mental health problems in the UK is an underdeveloped network of services designed to provide alternatives to inpatient care. Safe and effective interventions that can act as alternatives to inpatient admissions for CYP presenting in crisis are therefore highly desirable. Policymakers are increasingly recognizing this need, and in the UK strategies are in place to improve community mental health services [11]. NHS England typically commissions CAMHS specialist services through a competitive tendering process and involves several different stakeholders, including local authorities, providers, and patients and their families [12].

ICCS are a type of CAMHS specialist service for CYP with severe mental health problems that provide intensive treatment primarily outside of hospital in a community setting such as schools, homes, and religious or cultural centres [13]. ICCS are multidisciplinary teams of professionals, such as psychiatrists, psychologists, social workers, and nurses, who use a mix of evidence-based interventions to provide the best possible support to each child or young person, depending on their individual needs. These interventions may include supported discharge [14, 15], home treatment [16], intensive case management [17], assertive community treatment [18], and multisystemic therapy [19]. ICCS must meet minimum operational requirements across four themes [13]: (1) organizational boundaries (comply with admission criteria, adopt integrated models of care, provide out-of-hours support, active involvement in inpatient admission, timely and safe discharge), (2) human resources (caseload ratio of ≤ 10 service user to 1 provider, direct clinician contact per week of ≥ 2 episodes for at least 90% of caseload, team meetings ≥ 1 per week, monitoring of caseloads by ICCS lead, minimum ICCS team size of ≥ 4 FTE staff, clinical supervision of ≥ 1 per month for individual staff members, access to ≥ 1 psychiatrist or psychiatric prescriber), (3) nature and scope of services (≥ 80% of face-to-face contacts in community setting, advocating service user engagement with community resources, holistic approach to service user engagement, direct clinical contact for each service user on caseload of ≥ 2 h per week), and (4) evaluation (using mental health outcome measures to review and monitor service user progress). NHS-Led Provider Collaboratives are supporting the development of ICCS across England in line with the aims and investment priorities of Five Year Forward View for Mental Health to provide an alternative to admission to Tier 4 mental health facilities. Systematic reviews from 2009 [20], 2016 [21], and 2021 [22] did not find sufficient quality data to recommend a specific type of intervention as an alternative to inpatient admission for CYP presenting in mental health crisis. ICCS remains underutilized despite the accumulating evidence that it can provide a cost-effective alternative to inpatient care with similar clinical outcomes [23, 24].

### Objectives {7}

The primary trial objective is to evaluate the clinical effectiveness of ICCS compared to the treatment as usual (TAU) in improving the time taken for YP to return to or start EET, as measured by the number of days from randomization to the first day of attending EET. Secondary objectives are to (1) determine the relative effectiveness of ICCS in improving clinical outcomes and service satisfaction, (2) assess the relative cost-effectiveness of ICCS, (3) explore the experiences of CYP who receive ICCS, including their perceptions of the benefits and challenges of the intervention, and (4) explore the experiences of mental health professionals in delivering ICCS, including their perspectives on the strengths and weaknesses of the intervention.

### Trial design {8}

**Fig. 1:**
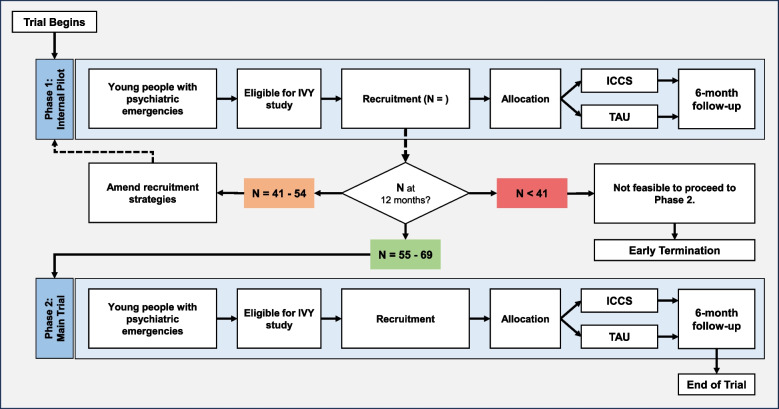
The trial schema shows the number of patients to recruit in Phase 1 (internal pilot) and the progression criterion to Phase 2 (main trial)

This is a non-commercial, multi-centre, parallel-group, single-blind (outcome assessor) randomized controlled superiority trial to test the hypothesis that young people randomized to ICCS will return to or gain EET significantly faster than young people who receive TAU when tested at 6 months post-randomization (a follow-up). A nested qualitative semi-structured interview study will explore service user experiences of inpatient care or ICCS treatment, and service provider experiences in delivering either ICCS or TAU. The trial was designed with two phases so that potential recruitment barriers within the first phase (an internal pilot) can be resolved before the second phase (the main trial). Progression to the main trial will depend on achieving projected recruitment targets within the first 12 months of the start of the RCT and will be reviewed with the trial steering committee (TSC) and the funder, National Institute for Health Research Health Technology Assessment Programme (NIHR-HTA). The trial schema is shown in Fig. 1. A traffic light system will be used to assess whether it is warranted and feasible to progress to the second phase:

- Green (go): progression to definitive trial if 80–100% of projected target is achieved.
- Amber (amend): review/amend recruitment strategies if 60–80% of the projected target is achieved.
- Red (stop): the study should not process as planned if < 60% of the projected target is achieved.

## Methods: participants, interventions and outcomes

### Study setting {9}

Participants will be recruited at nine centres participating in the trial: South London and Maudsley NHS Foundation Trust, East London NHS Foundation Trust, North East London NHS Foundation Trust, Central and North West London NHS Foundation Trust, Oxford Health NHS Foundation Trust, NHS Lothian, Berkshire Healthcare NHS Foundation Trust, Cambridgeshire and Peterborough NHS Foundation Trust, and Cwm Taf Morgannwg University Health Board. All centres will run the trial in accordance with good clinical practice guidelines and will work in collaboration with NIHR local research networks and with the King’s Clinical Trials Unit (KCTU).

### Eligibility criteria {10}

Inclusion and exclusion criteria for study participants are detailed in Table 1.

**Table 1:** Inclusion and exclusion criteria

Inclusion	Exclusion
1. Young people aged 12 years 0 months to 17 years 11 months (exclude if 18 at randomization) 2. Young people who can consent* and who are being considered for in-patient psychiatric admission or ICCS in the participating NHS Trusts. * Eligible participants under 16 years of age will require the consent of at least one person with parental responsibility	1. Local ICCS or TAU teams unable to accept new referrals due to their full capacity being reached. 2. Young people unable to consent due to their mental state. 3. The young person’s risk profile is incompatible with ensuring their safety and/or the safety of others in the community, as indicated by a Children’s Global Assessment Scale (CGAS) score of < 20

### Who will take informed consent? {26a}

Young people with psychiatric emergencies considered for inpatient admission within participating NHS CAMHS will be screened for trial suitability. The relevant clinical teams in each trust will explain the study to eligible participants and sufficient time will be given to read through the participant information sheet and ask questions, before deciding whether to take part. The trial research assistant from each centre will obtain consent and complete the baseline assessment prior to randomization.

### Additional consent provisions for collection and use of participant data and biological specimens {26b}

Not applicable. Participant data will not be used in ancillary studies and no biological specimens will be required in the study.

## Interventions

### Explanation for the choice of comparators {6b}

Participants randomized to the control arm will receive TAU, which may include inpatient care delivered in the hospital or all other community CAMHS except ICCS. After the initial assessment, young people are treated with a combination of psychological, pharmacological, and/or social interventions as needed to achieve the goals set with their families. Treatment is not time-limited, but the average duration of treatment is about 50 days. Hospital treatment is followed by standard community treatment.

### Intervention description {11a}

Participants randomized to the intervention arm will receive ICCS, which provides intensive treatment for young people with severe mental illness in a community setting. This study defines ICCS as consisting of the following essential components:

- Small caseload: ICCS teams will have a service user/provider ratio of no more than 10:1.
- Team approach: ICCS providers will function as a team rather than as individual practitioners, and clinicians will know and work with all clients.
- ICCS team meeting: ICCS teams will meet frequently to plan and review services for each service user.
- Practicing team leader: The supervisor of front-line clinicians will provide direct services.
- Continuity of staffing: ICCS teams will aim to maintain the same staffing over time.
- Staff capacity: ICCS teams will operate at full staffing.
- Psychiatrist/psychiatric prescriber on staff: There will be at least one full-time psychiatrist per 100 service users assigned to work with the ICCS team.
- Nurse (RMN) on staff: There will be at least two full-time nurses (RMNs) assigned to work with a 100-client ICCS team.
- ICCS team size: ICCS teams will be of sufficient absolute size to consistently provide the necessary staffing diversity and coverage.
- Explicit admission criteria: ICCS teams will have a clearly identified mission to serve a particular population and will have and use measurable and operationally defined criteria to screen out inappropriate referrals.
- Intake rate: ICCS teams will take clients in at a low rate to maintain a stable service environment.
- Responsibility for hospital admissions: ICCS teams will be involved in hospital admissions.
- Community-based services: ICCS teams will work to monitor status and develop skills in the community rather than function as office-based teams.
- No dropout policy: ICCS teams will engage and retain service users at a mutually satisfactory level.
- Assertive engagement mechanisms: ICCS teams will use community outreach, motivational/engagement techniques, as well as legal mechanisms or other techniques to ensure ongoing engagement.
- Intensity of service: ICCS teams will provide a high amount of face-to-face service time as needed.
- Frequency of contact: ICCS teams will provide a high number of face-to-face service contacts as needed.
- Work with informal support system: With or without service users present, ICCS teams will provide support and skills for service users’ support networks, such as family, school, and extracurricular activities coordinators.
- Role of service users on treatment team: Service users will be involved in the functioning of the team (e.g. as members of interview panels).
- Provision of a day service: ICCS teams will provide a form of day service, such as a day school or partial hospitalization, to those service users who need it.

After the initial assessment, individualized goals are set with the family. Treatment includes a combination of psychological, pharmacological, and/or social interventions as needed to achieve these goals. Interventions can be delivered up to several times a day. Treatment is not time-limited, but the aim is to achieve the goals within 3 months of the initial assessment. ICCS is followed by standard community treatment.

### Criteria for discontinuing or modifying allocated interventions {11b}

Service user-clinician partnership will tailor interventions to service user needs and preferences as per the engagement approach. Participants have the right to withdraw from the study at any time, without giving a reason. If a participant decides to withdraw, they will be asked on the same day to provide a reason for withdrawal, but they are not required to do so. If a participant withdraws from the study, we will make every effort to report the reason for withdrawal as thoroughly as possible (e.g. adverse events, inability to adhere, inability to attend regularly for treatment or assessment). For participants randomized to the intervention arm, ICCS will be discontinued if they no longer wish to continue, or the trial is terminated at the request of the Data Monitoring Committee (DMC). Randomized participants who wish to withdraw from ICCS will be asked to confirm whether they are still willing to provide study-specific data at a 6-month follow-up and if they wish to participate in the nested qualitative study. It is also possible that the clinical team might withdraw the participant from ICCS if allocated. The reason for a clinician’s decision to withdraw a patient from the study must be recorded and the relevant clinician will clinically assess the participant and arrange appropriate care. Researchers at each site will make every effort to obtain all outcome measures, in priority order, from participants who drop out of treatment as soon as possible. Reasons for and dates of withdrawal from the study will be recorded on a withdrawal form, which will describe the circumstances of the withdrawal. Safety concerns identified through adverse events (AEs) and serious adverse events (SAEs) will be addressed through protocol amendments.

### Strategies to improve adherence to interventions {11c}

Participant compliance with ICCS attendance will be supported by offering compensation for travel expenses, providing research appointment reminders and gift vouchers at baseline and 6-month follow-up. Participating sites will receive a study manual and links to video-based training on the clinician-administered semi-structured interview, the Kiddie-Schedule for Affective Disorders and Schizophrenia for School-Age Children-Present and Lifetime Version (K-SADSPL), and on a web-based electronic data capture solution (MACRO EDC) and the randomization procedures. All teams will be invited to a bi-weekly Q&A session for study support. Monthly study update meetings will be held for Principal Investigators (PIs) and research assistants from all sites.

### Relevant concomitant care permitted or prohibited during the trial {11d}

Participation in other studies is not an exclusion but will be assessed by the research team for appropriateness, i.e. participation in a research trial of another intervention designed to provide community support.

### Provisions for post-trial care {30}

Participants will remain under the clinical care of the service they are being treated in, which will arrange further treatment or discharge from the service according to their local practices.

### Outcomes {12}

#### Primary outcome

The primary outcome is a time-to-event endpoint, as measured by the number of days from randomization to the first day of returning to or starting education, employment, or training (EET). For participants who do not return to or start EET, the time will be censored at the end of the follow-up period (6 months after randomization) or at the time of consent withdrawal. The time to return to or start EET, regardless of the duration of attendance, will be collected by contacting the relevant clinical team or the relevant EET establishments. If a participant attends more than one establishment on the same date, this will be counted as one attendance. EET opportunities for young people are important outcome measures for social and economic policy, but they are also vital upstream determinants of health [25]. Young people who spend more time not in education, employment, or training (NEET) are more likely to experience poor physical and mental health, unemployment, low-quality work, and lower incomes later in life [26].

#### Secondary outcome

The following secondary outcome measures will be used to determine the impact of ICCS compared to TAU on the clinical symptoms, functioning and service satisfaction in young people: (1) The Strengths and Difficulties Questionnaire (SDQ), a self-reported measure of common areas of emotional and behavioural problems, will be administered at baseline and 6 months post-randomization. (2) The Children’s Global Assessment Scale (CGAS), a clinician-rated global measure of functioning in young people, will be administered at baseline and 6 months post-randomization. (3) The Clinical Global Impressions (CGI) and CGI Improvement Scales, a clinician-reported measure on their view of the patient’s global functioning before and after the study intervention, will be administered at pre-randomization and 6 months post-randomization, and the CGI Improvement Scale will be measured at 6 months post-randomization. (4) The ChASE children self-report questionnaire will be used to measure patient satisfaction with services at 6 months post-randomization. (5) The Health of the Nation Outcome Scales for Children and Adolescents (HoNOSCA), a clinician-completed scale that measures general health and social functioning, will be administered at baseline and 6 months post-randomization. (6) The Self-Harm Questionnaire, a self-reported measure of self-harm thoughts and behaviours, will be administered at baseline and 6 months post-randomization to identify participants who meet the diagnostic criteria for non-suicidal self-injury (NSI). (7) Length of stay in hospital defined by the time in days between hospital admission and discharge, will be collected from electronic patient records. (8) The number of days participants attend education, training, or employment in the 6 months following randomization will be collected by contacting the relevant clinical team or EET establishment. Independent researchers blinded to participants’ allocation will collect all outcomes at 6-month follow-up. We will mail families self-reported outcome measures with self-addressed envelopes or conduct telephone or remote interviews with families who prefer not to participate in face-to-face assessments. The following economic measures will be used to support assessment of the cost-effectiveness of ICCS compared to TAU: (1) Child Health Utility (CHU-9D), a self-reported measure of health-related quality of life in children and adolescents, will be administered at baseline and 6 months post-randomization. (2) The Child and Adolescent Service Use Schedule (CA-SUS), a measure of participant’s health and social care service resource-use data, will be collected at baseline and at follow-up to measure their service use 3 months prior to randomization and 6 months post-randomization, respectively. The CA-SUS will not include psychiatric inpatient, psychiatric day-patient and CAMHS services which will be collected from medical notes using a study-specific proforma. Semi-structured visual interviews will be conducted with 21 patients receiving ICCS, 21 patients receiving TAU, 21 mental health professionals delivering ICCS, and 21 mental health professionals delivering TAU. These interviews will be used to explore their self-reported experiences of receiving or providing care, respectively. A thematic decomposition analysis will be conducted to identify themes from descriptions of experience with ICCS and TAU, including its perceived strengths, barriers, facilitators, and challenges. As part of the existing process evaluation, we will also specifically investigate the outcomes that are most important to young people.

#### Data collection

Primary outcome (days to attend EET) will be requested and collected from the relevant establishment at the end of the follow-up period (6 months after randomization) or at study withdrawal. Sociodemographic data and a clinical diagnosis (K-SADS-PL) will be collected from participants prior to randomization. All secondary outcome measures will be assessed at baseline and at 6 months (± 1 month) after randomization, except for the CA-SUS measure of service use, which will only be assessed at 6 months. In the 6 months following the day of randomization, blinded research assistants will contact the relevant clinical teams to find out the first day the participant returned to or started EET, and their attendance record will be collected from the relevant educational establishments or employers. The opportunity to participate in a one-on-one semi-structured interview will be offered to young people and healthcare professionals to explore their experiences with ICCS and TAU. Research teams will transfer non-identifiable data captured on paper case report forms (CRFs) to a password-protected MACRO database within one month. To minimize data loss, completed CRFs will be scanned or copied first, and stored at the research team’s base (NHS Trust or university). All participant information will be kept confidential, with names and addresses removed. Identifiable data (consent forms, email addresses) will be kept in locked filing cabinets, separate from other research data.

#### Participant timeline {13}

The participant timeline is shown in Table 2.

**Table 2:**
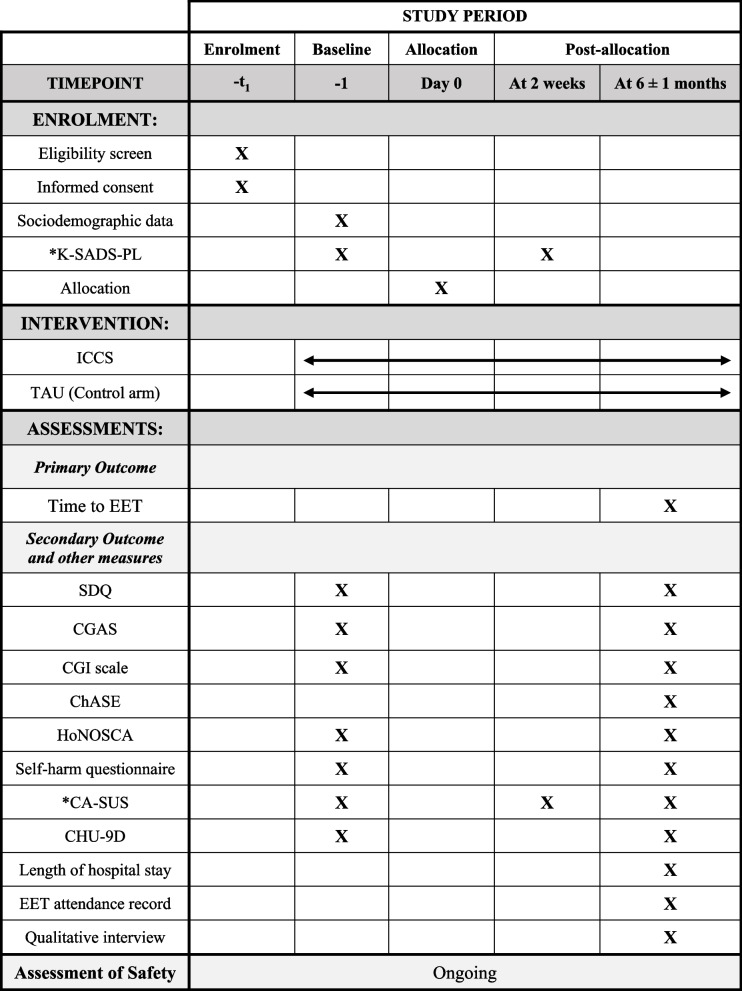
Schedule of enrolment, intervention and assessments

*K-SADS-PL and CA-SUS can be completed within 2 weeks of randomization

#### Sample size {14}

The sample size calculation is based on Ougrin et al (2018) [23] which estimated the proportion of young people not in employment, education or training (NEET) at 6 months follow-up as 49% for the control arm (TAU) and 19% for the intervention arm, indicating a difference of 30% (95% CI from 13.1 to 47.3%). In line with the Adult IAPT targets of achieving 50% recovery versus baseline 30% recovery [27], a reduction of 20% or larger in the proportion of young people NEET is clinically significant and therefore the minimum clinically significant (proportion) difference (MCID) is set to 20% (49% NEET under TAU and 29% NEET under ICCS), which is at the lower end of the CI for the effect size. An adjustment of 5% for loss to follow-up was used based on the findings from Ougrin et al (2014) [14]. A sample size of 252 participants, with 126 participants per trial arm, is required to detect a 20% reduction in the proportion of young people NEET with 90% power using a two-sided log-rank test. The sample size for the semi-structured qualitative interviews is based on recommendations for deciding saturation in theory-based interview studies. At least 42 young people will be recruited, 21 with experience of ICCS and 21 with experience of inpatient care. In addition to the service users, we will interview 42 mental health professionals delivering both intensive community care and inpatient care to examine their experience of care and treatment delivery.

#### Recruitment {15}

Each NHS Trust involved in the IVY trial was chosen based on patient availability and clinical expertise, among other criteria. Clinical teams from each site will identify young people with psychiatric emergencies considered for inpatient admission within NHS CAMHS. Clinically stable, potentially eligible patients and their families will be approached by the research assistant. A comprehensible and full explanation of the study with the aid of an age-appropriate Participant Information Sheet (PIS) will be given to the patient and their families. Informed consent will be obtained from young people aged 16–18 years. Assent will be provided by young people aged 12–15 years along with parents’/guardians’ consent.

#### Internal pilot

A 12-month pilot will assess whether enough eligible patients can be recruited in 12 months to make a trial viable within the proposed 36-month main trial recruitment period. To progress, the trial must recruit 55 patients in total. Recruiting 60–80% (41–54) of the projected recruitment will trigger a discussion with the funder to remedy recruitment difficulties. Recruiting less than 60% (< 41) will indicate that the trial should not proceed. The overall schema for the trial is shown in Fig. 1.

## Assignment of interventions: allocation

### Sequence generation {16a}

Participants will be randomized on a one-to-one basis to the intervention arm or control arm via the web-based KCTU randomization system using a computer-generated allocation sequence based on variable block sizes with stratification per NHS Trust.

### Concealment mechanism {16b}

Relevant members of the study team from each participating site will receive training and access to the KCTU randomization system. Allocation will be concealed using this computer-generated mechanism. The unblinded members of the study team will have no role in the outcome assessment. All accessors (research assistants) collecting follow-up outcomes will be blinded to the treatment allocation of the participants. The senior statistician will be blinded and the second statistician unblinded. All randomizations will be recorded in an associated KCTU database.

### Implementation {16c}

The unblinded study team member will obtain a unique Participant Identification Number (PIN) from the MACRO eCRF system and enter it with the stratification data into the KCTU system to randomize the participant and subsequently will notify the participant and their clinical team of the group allocation.

## Assignment of interventions: blinding

### Who will be blinded {17a}

The research assistants from each study site responsible for outcome assessment, the senior statisticians and the senior health economist will be blinded to group allocation.

### Procedure for unblinding if needed {17b}

Not applicable. There will be no circumstances during the trial under which unblinding of the outcome accessors will be required.

## Data collection and management

### Plans for assessment and collection of outcomes {18a}

Data will be collected at specified time points (see Table 2) using pre-designed case report forms, via telephone or follow-up clinic visits depending on the participant’s stated preference.

### Plans to promote participant retention and complete follow-up {18b}

To minimize loss to follow-up, we will implement a multifaceted approach that includes encouraging patients to discuss any difficulties with attending ICCS appointments with their treating clinicians, financial assistance towards travel for research-related appointments, appointment reminders, and other evidence-based strategies, such as using incentives, sending greeting cards, personalizing letters, using colour printing, and keeping measures short and easy to complete.

### Data management {19}

KCTU will set up and maintain a web-based electronic data capture (EDC) system using the MACRO (InferMed) software. The EDC will be tailored for this project and data will be collected on bespoke CRFs. The system is GCP and 21 CFR Part 11 compliant with full audit trail and database lock functionality. Relevant staff at participating trusts will be trained to ensure reliable data capture using a standard eCRF proforma. Proforma data will be recorded anonymously and monitored for completeness, then collated and entered consistently into the MACRO database by a blinded researcher at each site. Variable ranges in the eCRF will be limited to reduce data entry errors. The blinded research assistant will enter data collected after informed consent, and the trial management team will monitor data completeness and accuracy. Research data will be entered and stored electronically whenever possible. Hard copies will be stored in locked filing cabinets at NHS sites. Electronic copies will be password-protected and stored on secure shared drives. Research data will be archived at sites and centrally for up to 10 years, then shredded, deleted, or destroyed. Audio recordings from the qualitative interviews will be uploaded to secure shared drives in anonymised and encrypted form and then deleted from portable devices within 24 h. Data will be stored confidentially and securely, with anonymised outcome data stored separately from personal information. All data will be password-protected and transferred securely using encrypted zip files in CSV, Stata, SAS, or SPSS format. Data will not be shared outside of the project team and hosting organizations.

### Confidentiality {27}

The project will adhere to GCP and data protection, security, and confidentiality legislation, including the Caldicott Report, ISO IEC 27002, the Data Protection Act, the NHS Trust’s research policy, the NHS Research Governance Framework, and HRA/REC approval processes. In accordance with the General Data Protection Regulation (GDPR) and Data Protection Act 2018, all stored electronic data will be deidentified and stored on password-protected secure servers, only accessible to authorized research team members.

### Plans for collection, laboratory evaluation and storage of biological specimens for genetic or molecular analysis in this trial/future use {33}

This is not applicable as no biological specimens will be collected.

## Statistical methods

### Statistical methods for primary and secondary outcomes {20a}

We will conduct intention-to-treat (ITT) analyses according to a pre-specified statistical analysis plan (SAP) approved by the senior statistician and trial steering committee before database lock. Participants who do not return to EET will be right censored at the end of the follow-up period (6 months after randomization) or when consent is withdrawn. A Cox proportional hazards model with NHS Trust as a random intercept and a trust-varying random coefficient of the treatment pathway will be used to compare the primary time-to-event outcome (time to return to EET) between the two trial arms. To quantify the effect size a hazard ratio and associated 95% CI will be estimated. For continuous secondary outcomes, mixed regression modelling will be used to compare continuous secondary outcomes at 6 months between trial arms, with NHS Trusts as random intercepts and trust-varying random coefficients of treatment pathway. Corresponding baseline values will be included as covariates in the models, if available. Distributional assumptions will be checked, and nonparametric approaches used where necessary. The CGI variables will be compared between trial arms using mixed models with inferences generated by robust methods to avoid relying on distributional assumptions for these limited scale variables (e.g. robust standard errors or cluster bootstrap). For CGI (measured at both baseline and 6 months), the analysis model will include baseline values and treatment pathway as fixed effects and NHS Trust random effects (random intercept as well as random coefficients for treatment pathway). For CGI-I (measured only at 6 months), models will not have a corresponding baseline value. Secondary outcomes measured as days on which an event happens over an observation period (e.g. time spent in hospital, number of days attending EET in the 6 months after randomization) will be compared between trial arms using a binomial distribution with a logit link and will allow for overdispersion. We will check whether the binomial distribution can be approximated by a Poisson model with overdispersion (i.e. negative binomial distribution, including an offset for the observation period). The models will contain the same terms as previous models, and the effect will be expressed as odds ratios (binomial distribution) or incidence rate ratios (Poisson model), depending on the distributional assumptions made. Binary outcomes from the Self-Harm Questionnaire will be analysed using mixed-effects logistic regression to compare the odds of participants reporting multiple (5 or more) episodes of self-harm between trial arms. Models will include baseline values and trial arm as fixed effects, and NHS Trust as a random intercept and random coefficient. Trial arm effect sizes will be reported with 95% confidence intervals (CIs). *P*-values will be presented, but there are no pre-specified significance thresholds for the (multiple) secondary outcomes.

The health economic analysis will use a within-trial, intention-to-treat approach with all randomized participants and taking the NHS/Personal Social Services perspective, including health and social care services provided with the education sector. As participants are in crisis at study entry (baseline), resource use data will be collected using a brief version of the CA-SUS focused on key health care resources, with a two-week window for completion. At 6-month follow-up, we will collect all-cause health and social care resources utilized by participants, excluding psychiatric inpatient, day-patient, and CAMHS services to avoid unblinding of research assessors. Instead, information on the use of these mental health services will be collected from clinical records at each site using a proforma to ensure accuracy and minimize unblinding.

Services will be costed using the most up-to-date nationally applicable unit costs [28]. Our primary economic analysis will be a cost-utility analysis using quality-adjusted life years (QALYs) derived from the CHU9D, with appropriate utility weights attached to health states [29] and QALYs calculated using the total area under the curve approach with linear interpolation between assessment points [30]. We will conduct a secondary cost-effectiveness analysis using the primary clinical outcome measure (time to EET). Costs and outcomes will be compared by trial arm, with mean values and standard deviations presented. Non-parametric bootstrap regressions will be used to obtain mean differences in costs and 95% confidence intervals, accounting for the non-normal distribution of economic data [31]. Cost-effectiveness will be assessed using the net benefit approach and standard methods [32]. A joint distribution of incremental mean costs and effects will be generated using bootstrapping to explore the probability of ICCS being the optimal choice compared to TAU, subject to a range of possible maximum values (ceiling ratio) that a decision-maker might be willing to pay for unit improvements in outcomes. Cost-effectiveness acceptability curves will be presented by plotting these probabilities for a range of possible values of the ceiling ratio [33]. These curves are a recommended decision-making approach for dealing with uncertainty in cost and effect estimates, and uncertainty regarding the maximum cost-effectiveness ratio that a decision-maker would consider acceptable. To provide more relevant treatment-effect estimates, all economic analyses will be adjusted for pre-specified variables of interest and baseline covariates, in line with the clinical analyses [34].

### Interim analyses {21b}

This is not applicable. No interim analyses will be conducted because all follow-ups occur at 6 months post-randomization and therefore will not inform intervention modification or discontinuation. The sponsor or chief investigator may prematurely discontinue the trial due to new safety information, lack of recruitment, or other reasons approved by the data monitoring committee (DMC), trial steering committee (TSC), regulatory authority, or ethics committee.

### Methods for additional analyses (e.g. subgroup analyses) {20b}

There are no planned subgroup analyses.

### Methods in analysis to handle protocol non-adherence and any statistical methods to handle missing data {20c}

Information on the time to return to EET will be collected throughout the follow-up period, up to 6 months after randomization. Secondary outcomes will be measured at 6-month follow-up and at baseline where specified. We will report the number and proportion of participants with complete data. If there is no published guidance on handling missing values for included measures of outcome, we will prorate the scales/subscales (i.e. calculate the average value of the complete items and use it to replace the missing values, if no more than 20% of the items are missing). Missing baseline data is not an issue for the primary analysis, as NHS Trust is the only covariate. If there are missing items at baseline for secondary outcomes, mean imputation may be applied, as recommended by White and Thompson [35]. The primary analysis of time/return to EET is expected to have relatively complete data, as the dates will be requested from clinical teams and institutions, rather than participant interviews. For secondary outcomes with > 10% missing data at 6-month follow-up, we will investigate baseline predictors of missingness and include them in adjusted models, such as conditioning or multiple imputation. Participants in both arms are expected to receive an adequate dose of treatment, although the implementation of TAU may vary between trusts.

### Plans to give access to the full protocol, participant level-data and statistical code {31c}

The full protocol is available on the funder’s website. To ensure comprehensive and transparent reporting of study findings, we will adhere to the CONSORT-SPI 2018 Extension reporting guidelines [36]. Participant flow through the study will be depicted using a CONSORT-SPI flow diagram, including the number of participants approached for enrolment, the decline rate, and assessments to confirm eligibility and outcomes between groups. To prevent selective reporting, all outcomes will be reported as specified in this study protocol. The study team will have exclusive use of the data until all planned primary and secondary analyses are published. After that, the anonymized quantitative dataset will be available from the corresponding author upon reasonable request.

## Oversight and monitoring

### Composition of the coordinating centre and trial steering committee {5d}

The trial management group (TMG) will consist of the chief investigator (CI), trial manager, research assistants, and trial statistician team. The CI, trial manager, and research assistants will meet weekly to ensure that the study is progressing according to the protocol, planned timelines, and budget. The trial steering committee (TSC) will be appointed and function according to HTA guidelines. The TSC will meet twice yearly to provide expert oversight and advice on all aspects of the trial. The TMG will prepare a report for the TSC.

### Composition of the data monitoring committee, its role and reporting structure {21a}

The DMC will be appointed and function according to HTA guidelines. The DMC will meet twice in the first and second years of the project and function according to HTA standard operating procedures. An independent chair and statistician will be appointed to the DMC. The DMC will monitor initial patient recruitment numbers (i.e. numbers recruited at the sites). The DMC will advise on recruitment difficulties and resource allocation.

### Adverse event reporting and harms {22}

Clinical teams will monitor safety throughout the study. Any unfavourable or unintended sign, symptom, or illness (AE) will be recorded, including exacerbations of pre-existing illnesses, increased frequency or intensity of pre-existing episodic events or conditions, conditions detected after randomization, and continuous persistent disease or symptoms present at baseline that worsen following randomization. Serious AEs (SAEs) include life-threatening events, death, inpatient hospitalization or prolongation of existing hospitalization, significant or persistent incapacity/disability, or pregnancy. We will report AEs from the signing of the study consent form to the last follow-up assessment 6 months after randomization. The chief investigator and an independent clinical reviewer will assess the causality and expectedness of SAEs. We will submit serious, related and expected (SAR) events to the DMEC/TSC and sponsor as needed. We will report all related and unrelated SAEs to the participant's NHS Trust and the REC as appropriate. We will also report any events rated as also unexpected (SUSAR) to the REC within 15 days of first notice.

### Frequency and plans for auditing trial conduct {23}

The investigator(s) will grant direct access to source data and other documents to the sponsor(s), REC, authorized representatives of the sponsor, NHS, regulatory authorities, and RECs for trial-related monitoring, audits, and review.

### Plans for communicating important protocol amendments to relevant parties (e.g. trial participants, ethical committees) {25}

We will obtain sponsor approval for all protocol amendments, submit substantial amendments to the REC for written approval, and communicate all amendments to participating NHS Trusts. Amendments will be updated on the clinical trial registry, but not communicated to participants. We will submit an annual progress report to the REC, HRA (where required), sponsor, and funder, and an end-of-study notification and final report to the same parties.

### Dissemination plans {31a}

We will use a multi-modal dissemination plan to share the trial’s outcomes with academics, CAMHS staff, service users, and other stakeholders. We will publish the findings in academic papers, present them at conferences, and share them via social media, newsletters, events, and charities. We will also inform professional bodies, the National Institute for Health and Care Excellence (NICE), NHS England, and equivalent bodies in Scotland and Wales.

## Discussion

Community-based treatment and inpatient admission are distinct models of care. The existing research is not sufficient to determine their relative effectiveness to enable evidence-informed policymaking and service planning. The IVY study is the only study to date with substantial potential to address the uncertainties in the true clinical and cost-effectiveness of ICCS as an alternative to inpatient admission. The IVY study is a definitive RCT in a real-life healthcare setting, at multiple sites with a representative geographical spread and a range of clinicians from diverse disciplinary backgrounds. We chose time to EET as the primary outcome measure because economic studies have shown that achieving both health and employment outcomes is necessary to generate better social and economic benefits for individuals and society [37]. This outcome measure is also compatible with the National Mental Health Service Dataset, making the trial results highly compelling to policymakers, healthcare providers, and mental health researchers. The trial results could lead to positive changes in clinical practice, such as developing new guidelines. The IVY study will evaluate ICCS's impact on a wide range of symptomatology and health outcomes experienced by young people with psychiatric emergencies, including self-harm, suicidality, general health, and well-being. The study will also provide accurate information about the services accessed under ICCS and TAU. A comprehensive cost-effectiveness evaluation will assess costs offset and costs saved by ICCS compared to TAU. Thus, the IVY study will provide unprecedented data on the relative clinical effectiveness, cost-effectiveness, and service utilization of ICCS and TAU for young people with psychiatric emergencies.

### Trial status

The current protocol is version 2.7, dated August 10, 2023. The IVY study started in October 2020 and was paused from March 2020 until the end of September 2021 due to the coronavirus pandemic. COVID-19 caused a pause in a large proportion of NHS research, including the IVY study, which was also affected by the closure of UK schools, as the primary efficacy parameter of the study is time to EET. Site activation was staggered with sites reopening for recruitment 6 months after project restart, due to a backlog in internal review processes from disruptions caused by the pandemic and staff shortages. SAP for this trial was approved by the TSC chair in November 2022. Recruitment restarted in February 2022, but was stopped in September 2023 due to not meeting the progression criteria for the internal pilot. Patient follow-up is ongoing, with the final follow-up visit scheduled for February 2024.

## Abbreviations

AE: Adverse event
CAMHS: Child and Adolescent Mental Health Services
CHU9D: Child Health Utility
CRF: Case report form
CYP: Children and young people
DMC: Data Monitoring Committee
EDC: Electronic data capture
EET: Education, employment or training
GCP: Good clinical practice
ICCS: Intensive Community Care Services
ITT: Intention to treat
KCTU: King’s Clinical Trials Unit
PPI: Patient and Public Engagement
QALY: Quality-adjusted life year
RCT: Randomized controlled trial
REC: Research Ethics Committee
SAP: Statistical analysis plan
SAE: Serious adverse event
TAU: Treatment as usual
TMG: Trial management group
TSC: Trial steering committee
YP: Young people

## References

[1] Newlove-Delgado T MF, Williams T, Mandalia D, Davis J, McManus S, Savic M, Treloar W, Ford T. : Mental Health of Children and Young People in England, 2022. In. Edited by NHSDigital. Leeds; 2022.

[2] NHS Digital. National Estimates, August 2021 to March 2022. England: NHS Digital; 2022.

[3] Green J, Jacobs B, Beecham J, Dunn G, Kroll L, Tobias C, Briskman J (2007). Inpatient treatment in child and adolescent psychiatry--a prospective study of health gain and costs. J Child Psychol Psychiatry..

[4] Stewart SL, Semovski V, Lapshina N (2022). Adolescent Inpatient Mental Health Admissions: An Exploration of Interpersonal Polyvictimization, Family Dysfunction, Self-Harm and Suicidal Behaviours.

[5] Miller DAA, Ronis ST, Slaunwhite AK, Audas R, Richard J, Tilleczek K, Zhang M (2020). Longitudinal examination of youth readmission to mental health inpatient units. Child Adolesc Ment Health..

[6] DHSC: Reforming the Mental Health Act : Government response to consultation. In: UK Parliament Command Paper, session 2021/22 CP 501. Edited by Care DoHaS. Crown Copyright, London; 2021.

[7] Care Quality Commission: The state of health care and adult social care in England 2021/22. Crown Copyright, London; 2022.

[8] Worrall A, O'Herlihy A, Banerjee S, Jaffa T, Lelliott P, Hill P, Scott A, Brook H (2004). Inappropriate admission of young people with mental disorder to adult psychiatric wards and paediatric wards: cross sectional study of six months’ activity. BMJ..

[9] Wong BH, Cross S, Zavaleta-Ramirez P, Bauda I, Hoffman P, Ibeziako P, Nussbaum L, Berger GE, Hassanian-Moghaddam H, Kapornai K (2023). Self-Harm in Children and Adolescents Who Presented at Emergency Units During the COVID-19 Pandemic: An International Retrospective Cohort Study. J Am Acad Child Adolesc Psychiatry..

[10] Grimm FAB, Butler J, Fernandez Crespo R, Davies A, Peytrignet S, Piroddi R, Thorlby R, Tallack C (2022). Improving children and young people’s mental health services: Local data insights from England.

[11] Alderwick H, Dixon J (2019). The NHS long term plan. BMJ..

[12] Gongora-Salazar P, Glogowska M, Fitzpatrick R, Perera R, Tsiachristas A (2022). Commissioning [Integrated] Care in England: An Analysis of the Current Decision Context. Int J Integr Care..

[13] Keiller E, Masood S, Wong BH, Avent C, Bediako K, Bird RM, Boege I, Casanovas M, Dobler VB, James M (2023). Intensive community care services for children and young people in psychiatric crisis: an expert opinion. BMC Med..

[14] Ougrin D, Zundel T, Corrigall R, Padmore J, Loh C (2014). Innovations in Practice: pilot evaluation of the supported discharge service (SDS): clinical outcomes and service use. Child Adolesc Ment Health..

[15] Ougrin D, Corrigall R, Stahl D, Poole J, Zundel T, Wait M, Slater V, Reavey P, Byford S, Ivens J (2021). Supported discharge service versus inpatient care evaluation (SITE): a randomised controlled trial comparing effectiveness of an intensive community care service versus inpatient treatment as usual for adolescents with severe psychiatric disorders: self-harm, functional impairment, and educational and clinical outcomes. Eur Child Adolesc Psychiatry..

[16] Boege I, Corpus N, Weichard M, Schepker R, Young P, Fegert JM (2021). Long-term outcome of intensive home treatment for children and adolescents with mental health problems - 4 years after a randomized controlled clinical trial. Child Adolesc Ment Health..

[17] Dieterich M, Irving CB, Bergman H, Khokhar MA, Park B, Marshall M (2017). Intensive case management for severe mental illness. Cochrane Database Syst Rev..

[18] Mantzouranis G, Baier V, Holzer L, Urben S, Villard E (2019). Clinical significance of assertive community treatment among adolescents. Soc Psychiatry Psychiatr Epidemiol..

[19] Henggeler SW, Rowland MD, Halliday-Boykins C, Sheidow AJ, Ward DM, Randall J, Pickrel SG, Cunningham PB, Edwards J (2003). One-year follow-up of multisystemic therapy as an alternative to the hospitalization of youths in psychiatric crisis. J Am Acad Child Adolesc Psychiatry..

[20] Shepperd S, Doll H, Gowers S, James A, Fazel M, Fitzpatrick R, et al. Alternatives to inpatient mental health care for children and young people. Cochrane Database Syst Rev. 2009;(2):CD006410. 10.1002/14651858.CD006410.pub2 PMC4014676 19370634

[21] Kwok KHR, Yuan SNV, Ougrin D (2016). Review: Alternatives to inpatient care for children and adolescents with mental health disorders. Child Adolesc Ment Health..

[22] Clisu DA, Layther I, Dover D, Viner RM, Read T, Cheesman D, Hodges S, Hudson LD (2022). Alternatives to mental health admissions for children and adolescents experiencing mental health crises: A systematic review of the literature. Clin Child Psychol Psychiatry..

[23] Ougrin D, Corrigall R, Poole J, Zundel T, Sarhane M, Slater V, Stahl D, Reavey P, Byford S, Heslin M (2018). Comparison of effectiveness and cost-effectiveness of an intensive community supported discharge service versus treatment as usual for adolescents with psychiatric emergencies: a randomised controlled trial. Lancet Psychiatry..

[24] Boege I, Corpus N, Schepker R, Kilian R, Fegert JM (2015). Cost-effectiveness of intensive home treatment enhanced by inpatient treatment elements in child and adolescent psychiatry in Germany: A randomised trial. Eur Psychiatry..

[25] Hale DR, Bevilacqua L, Viner RM (2015). Adolescent Health and Adult Education and Employment: A Systematic Review. Pediatrics..

[26] Clayborne ZM, Varin M, Colman I (2019). Systematic Review and Meta-Analysis: Adolescent Depression and Long-Term Psychosocial Outcomes. J Am Acad Child Adolesc Psychiatry..

[27] Clark DM (2018). Realizing the Mass Public Benefit of Evidence-Based Psychological Therapies: The IAPT Program. Annu Rev Clin Psychol..

[28] Jones K, Weatherly, H. L. A., Birch, S., Castelli, A., Chalkley, M. J., Dargan, A., Forder, J., Gao, M., Hinde, S., Markham, S., Ogunleye, D., Premji, S., & Roland, D: Unit Costs of Health and Social Care 2022. In., 2022 edn. Kent: ersonal Social Services Research Unit, University of Kent at Canterbury; 2022: 121.

[29] Stevens K (2012). Valuation of the Child Health Utility 9D Index. Pharmacoeconomics..

[30] Manca A, Hawkins N, Sculpher MJ (2005). Estimating mean QALYs in trial-based cost-effectiveness analysis: the importance of controlling for baseline utility. Health Econ..

[31] Thompson SG, Barber JA (2000). How should cost data in pragmatic randomised trials be analysed?. BMJ..

[32] Briggs AH (1999). A Bayesian approach to stochastic cost-effectiveness analysis. Health Econ..

[33] Fenwick E, Byford S (2005). A guide to cost-effectiveness acceptability curves. Br J Psychiatry..

[34] Assmann SF, Pocock SJ, Enos LE, Kasten LE (2000). Subgroup analysis and other (mis) uses of baseline data in clinical trials. Lancet..

[35] White IR, Thompson SG (2005). Adjusting for partially missing baseline measurements in randomized trials. Stat Med..

[36] Montgomery P, Grant S, Mayo-Wilson E, Macdonald G, Michie S, Hopewell S, Moher D, Group C-S (2018). Reporting randomised trials of social and psychological interventions: the CONSORT-SPI 2018 Extension. Trials..

[37] Marmot M (2020). Health equity in England: the Marmot review 10 years on. BMJ..

